# Integration of bulk RNA sequencing data and single-cell RNA sequencing analysis on the heterogeneity in patients with colorectal cancer

**DOI:** 10.1007/s10142-023-01102-3

**Published:** 2023-06-24

**Authors:** Jiawei Zhang, Yangsheng Wu, Zhong Shen

**Affiliations:** 1 The Second Clinical Medical College, Zhejiang Chinese Medical University, Hangzhou, 310053 China; 2 College of Life Science, Zhejiang Chinese Medical University, Hangzhou, 310053 China; 3 Department of Coloproctology, The Hangzhou Third People’s Hospital, the No.38 Westlake Avenue, Hangzhou City, 310009 Zhejiang Province China

**Keywords:** Colorectal cancer, scRNA-seq, Molecular subtypes, Immune, Prognosis

## Abstract

The cyclic GMP-AMP synthase (cGAS)-stimulator of interferon genes (STING) pathway has emerged as a critical innate immune pathway that could virtually impact nearly all aspects of tumorigenesis including colorectal cancer. This work aimed to develop and validate molecular subtypes related to cGAS-STING pathways for colorectal cancer using Bulk RNA-seq and single-cell RNA-seq (scRNA-seq) data. Bulk RNA-seq data were acquired from The Cancer Genome Atlas dataset (training dataset) and Gene Expression Omnibus dataset (validation dataset). Univariate COX survival analysis was utilized to identify prognostic differentially expressed genes (DEGs) from 6 immune pathways related to cGAS-STING. ConsensusClusterPlus package was used to classify different subtypes based on DEGs. scRNA-seq data were used to validate differences in immune status between different subtypes. Two clusters with distinct prognosis were identified based on 27 DEGs. The six cGAS-STING-related pathways had different levels of significance between the two clusters. Clust1 had most number of amplified CNVs and clust2 had the most number of loss CNVs. *TP53* was the top mutated gene of which missense mutations contributed the most of single-nucleotide variants. Immune score of clust1 was higher than that in clust2, as reflected in macrophages, T cells, and natural killer cells. Three unfavorable genes and 31 protection factors were screened between the two clusters in three datasets. ScRNA-seq data analysis demonstrated that macrophages were more enriched in clust1, and tumor cells and immune cells had close interaction. We classified two distinct subtypes with different prognosis, mutation landscape, and immune characteristics.

**Supplementary Information:**

The online version contains supplementary material available at 10.1007/s10142-023-01102-3.

## Introduction

Colorectal cancer is the third most common cancer in the world and the second most fatal cancer for humans (Siegel et al. 2020). The 5-year survival rate for colorectal cancer is about 64%, which reduce to 14% after metastasis (Siegel et al. 2020). Therefore, there is an urgent need to develop new therapeutic targets. Due to the heterogeneity of colorectal cancer, the treatment of the cancer is not a simple medical process. The pathogenesis and evolution of colorectal cancer have not been fully understood, and the research on the treatment of colorectal cancer and development of related drugs is still challenging. Therefore, we hope that the research on colorectal cancer subtypes can provide more theoretical basis for treating colorectal cancer.

A cancer can be divided into different subtypes due to different genes that cause it. At present, research on cancer subtypes has been widely studied. Sadanandam et al. defined six clinically relevant colorectal cancer subtypes by analyzing the gene expression profiles of 1290 colorectal cancer patients using consistent clustering (Sadanandam et al. 2013). Still with unsupervised clustering, based on the genome-wide data from 188 colorectal cancer patients, Roepman et al. identified three major subtypes (type A, B, and C) that were validated in 543 patients with stage II to III, and the subtypes were associated with prognosis and degree of benefit from chemotherapy (Roepman et al. 2014). Felipe et al. used unsupervised clustering to define three major subtypes among 1100 patients with colorectal cancer (De Sousa et al. 2013).

The cyclic GMP-AMP synthase (cGAS)-stimulator of interferon genes (STING) pathway is a cytosolic double-stranded DNA sensor of the innate immune system, which is important in the response to pathogen infection and inflammation. In addition, the cGAS-STING pathway is responsible for the innate immune recognition of cancer. Thus, it plays a critical role in anti-cancer immunity and enhances the effects of cancer immunotherapies (Jiang et al. 2020). Furthermore, Kaneta et al. have recently shown that downregulation of DNA mismatch repair genes promotes the activation of the cGMP-STING pathway, which is important for the recruitment of CD8 + cells into the tumor microenvironment of colorectal cancer (Kaneta et al. 2022).

In recent years, single-cell RNA-seq (scRNA-seq) was used to quantify expression in different cell populations, enabling researchers to analyze differences among cells (Yip et al. 2019; Esaulova et al. 2020). Complete characterization of single cell transcriptional landscape has great potential in detecting clinically important tumor subsets, understanding tumor heterogeneity and clinical application (Peng et al. 2019; Bao et al. 2021). Clustering of single-cell expression data helps identify cell types from a large number of heterogeneous cells and can be used for multiple downstream expression analyses (Yip et al. 2019). Hence, traditional analysis of colorectal cancer based on bulk RNA-seq would be quite insufficient.

This study identified the clustering subtypes of colorectal cancer based on the characteristics of cGAS-STING-related pathways, and correlated the characteristics of each subtype with patients’ prognosis, gene mutation, immune status, and immune cell infiltration. In addition, single-cell RNA sequence (scRNA-seq) was used to identify potential cell subtypes of colorectal cancer and elucidate the role of malignant cells in the tumor microenvironment. Our findings provided new insights into the prognostic characteristics of colorectal cancer and will contribute to the development of effective immunotherapy strategies for colorectal cancer.

## Material and methods

### Raw data acquisition

Public colorectal cancer bulk RNA-seq data, clinicopathological characteristics, and mutation data (CNV and SNP) were retrieved from The Cancer Genome Atlas (TCGA) database (https://portal.gdc.cancer.gov/) (Jager et al. 2018) and Gene Expression Omnibus (GEO) database (https://xena.ucsc.edu/) (Toro-Domínguez et al. 2019) (GSE17536 (Xu et al. 2020) and GSE17538 (Chen et al. 2021)). A total of 431 samples in total from the TCGA-colorectal cancer cohort and 177 samples in the GSE17536 cohort, and 238 samples in the GSE17538 cohort, were used in further analysis.

The colorectal cancer scRNA-seq dataset (GSE161277) was downloaded from the GEO database and included 13 samples. The raw data contained a total of 50,061 cells. The percentage of mitochondria and rRNA was calculated applying the PercentageFeatureSet function. Genes expressed in each cell were more than 500 and less than 7000, and mitochondrial content was less than 25%. In addition, the number of UMIs in each cell was at least 100 but less than 500. The number of cells after filtration was 26961.

### Molecular signatures database analysis

The comprehensive database molecular signatures database (MSigDB) (www.gsea-msigdb.org/gsea/msigdb/) (Liberzon et al. 2015) includes > 10,000 gene sets and is widely used to perform gene set enrichment analysis. In this work, we used this database to obtain genes (370 genes) related to 6 cGAS-STING pathways (Yang et al. 2021a) (APOTOSIS, B cell receptor signaling pathway, Chemokine signaling pathway, RIG-I-like-receptor signaling pathway, T cell receptor signaling pathway, and Toll-like receptor signaling pathway).

### Univariate COX survival analysis

In the TCGA dataset, univariate cox analysis was performed on the 370 genes in the 6 cGAS-STING pathways by the COXPH function of the survival package (Zhang 2016) under the threshold of *p* < 0.05.

### Identification of molecular subtypes

Based on univariate COX survival analysis, prognostic genes were obtained, and ConsensusClusterPlus package (Wilkerson and Hayes 2010) was used to cluster 431 samples in the TCGA-cancer cohort. At the same time, we used PAM algorithm and Pearson as a measure of distance and 500 bootstraps. The number of clusters K was set as 2 ~ 10, and the best classification was determined by calculating the consistency matrix and cumulative distribution function. Principal component analysis (PCA) (Casal et al. 2021) was introduced to support heterogeneity between subtypes.

### Single-sample GSEA

Single-sample GSEA (ssGSEA) analysis was performed in the GSVA package (Hänzelmann et al. 2013) to obtain a hallmark gene set score and the Hallmark gene set was obtained from MSigDB. Wilcox.test was conducted to evaluate the differences of 6 cGAS-STING pathway score, epithelial-mesenchymal transition (EMT) score, Toll-like receptor score, natural killer (NK) cytotoxicity score, and antigen processing and presentation score between the molecular subtypes.

### Clinical relevance and mutation landscape between molecular subtypes

The association of clinicopathological characteristics of patients in the TCGA cohort between molecular subtypes was analyzed using chi-square test. Gistic2 (Roufas et al. 2021) was conducted for copy number variation (CNV) of patients in the TCGA-colorectal cancer cohort with confidence level of 0.9 and hg38 as reference genome. In addition, waterfall plot was generated to explore detailed single-nucleotide variant (SNV) characteristics between molecular subtypes via “oncoplot” function in R software, “maftools” package (Mayakonda et al. 2018).

### Cell-type identification by estimating relative subsets of RNA transcripts

Cell-type identification by estimating relative subsets of RNA transcripts analysis was used to compare differences in various immune cells in molecular subtypes. Wilcox.test analysis was performed to determine the difference of 22 types of infiltrating immune cells score between molecular subtypes. The “ggplot2” package (Ito and Murphy 2013) was employed to visualize the distribution of the differences in 22 types of infiltrating immune cells.

### Calculation of ImmuneScore, StromalScore, and EstimateScore

R software ESTIMATE algorithm (Yang et al. 2021b) was used to calculate overall stromal content (StromalScore), immune infiltration (ImmuneScore), and combined (ESTIMATEScore) of patients from the TCGA-colorectal cancer cohort using Wilcox.test analysis to determine difference between the molecular subtypes.

### Differential expressed genes analysis

Differential expressed gene (DEG) analysis was performed on subtypes of TCGA-cancer cohort, GSE17536 cohort, and GSE17538 cohort, respectively, using limma package (Ritchie et al. 2015) with the cutoff of |log2FoldChange (logFC)|> log2(1.2) and FDR < 0.05. ‘ggplot2’ and ‘venn’ packages (Gao et al. 2021) were applied to generate volcano plots and venn plots, respectively.

The gene–gene interaction was screened by STRING (https://cn.string-db.org/) (Szklarczyk et al. 2021) with score ≥ 0.4, and visualized by Cytoscape (3.8.0) (Otasek et al. 2019).

### ScRNA-Seq data analysis

PCA was performed on the 2000 genes, and uniform manifold approximation and projection was used for dimensionality reduction and cluster identification (Becht et al. 2018). “FindNeighbors” and “FindClusters” function was used to cluster the cells (Resolution = 0.2). The “Find All Markers” function was employed to identify obvious marker genes for different clusters by setting log2 (FC) as 0.5 and Minpct as 0.5. ClusterProfiler package (Yu et al. 2012) was used in Kyoto Encyclopedia of Genes and Genomes (KEGG) analysis. Next, Copykat package were used to predict the CNV of cells to distinguish between tumor cells and normal cells. Finally, cell–cell communication analysis and network visualization was performed using R software, “CellChat” (Jin et al. 2021) and “patchwork” packages.

### Statistical analysis

R software (version 4.0.3) were used for statistical analysis. Kaplan–Meier survival curves were plotted for survival analysis by the survminer R package version 2.43–3. A *P*-value < 0.05 indicated a statistical significance (*P < 0.05; **P < 0.01; ***P < 0.001; ****P < 0.0001).

## Results

### Two molecular subtypes were identified based on cGAS-STING-related pathways

**Fig. 1:**
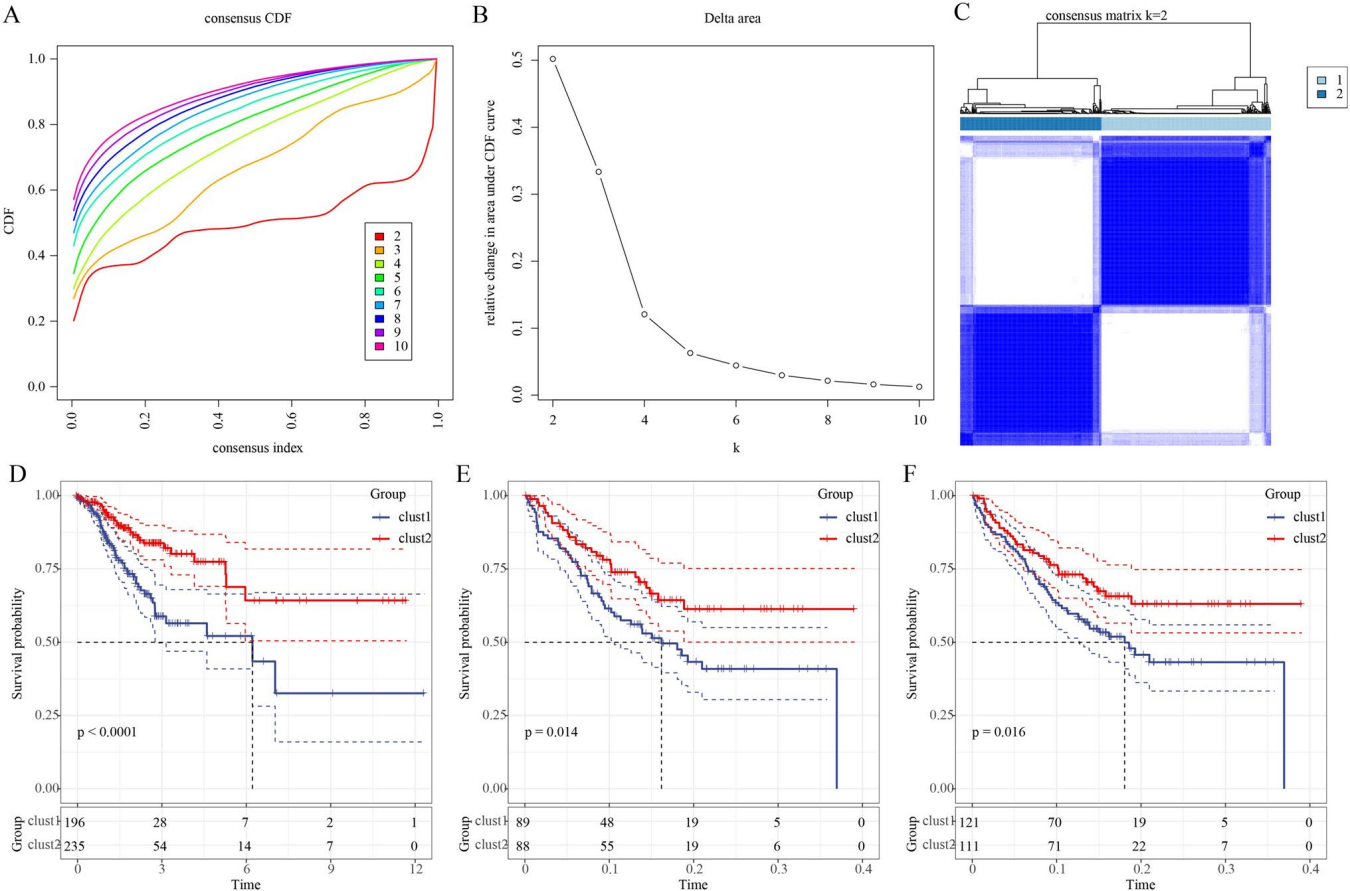
Two clusters were identified in TCGA-colorectal cancer. **A** Cumulative distribution function (CDF) curve of TCGA-colorectal cancer. **B** Under cumulative distribution function (CDF) curve. **C** Consensus *k* = 2, TCGA-colorectal cancer samples were divided into two clusters. **D**–**F** Kaplan–Meier prognosis curves of two clusters respectively in TCGA-colorectal cancer, GSE17536 dataset, and GSE17538 dataset

**Fig. 2:**
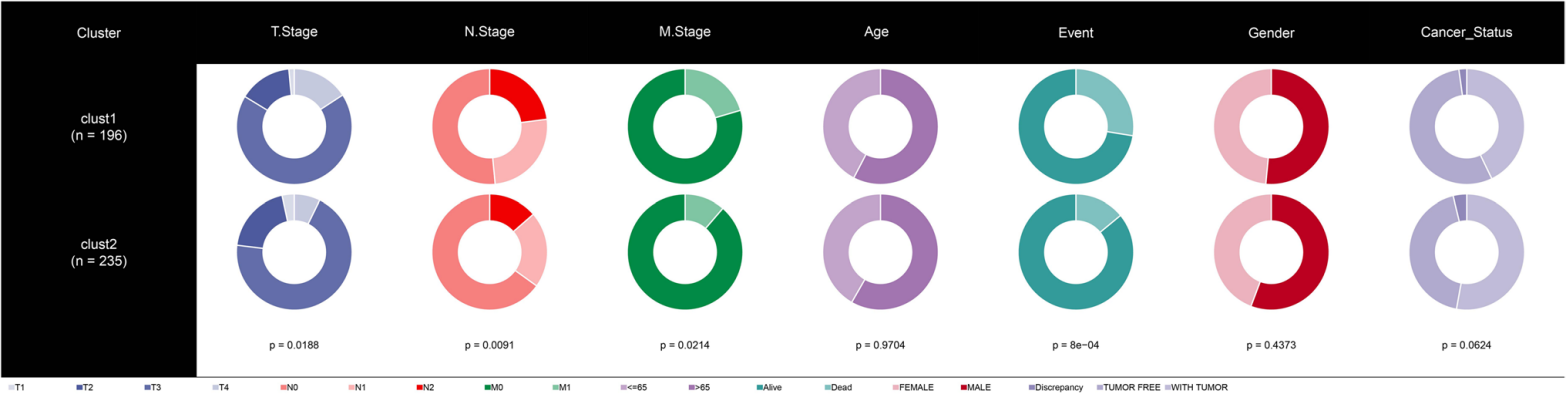
Difference distribution of clinical features between the two clusters in TCGA-colorectal cancer

In TCGA-COAD dataset, genes in cGAS-STING-related pathways (six pathways) were screened using univariate Cox proportional hazards survival analysis, and we found 27 genes were associated with prognosis (Table 1). Based on these 27 genes, 431 COAD samples were classified by ConsensusClusterPlus; as a result, Cluster number *k* = 2 was confirmed as the optimal according to CDF and area under CDF curve (Fig. 1A, B). Consensus matrix showed that the samples were clearly divided into two molecular subtypes (clusters, Fig. 1C). Similar results were observed in GEO dataset (Figure S1). Kaplan–Meier survival analysis revealed that the patients in cluster 2 had longer survival time in comparison to cluster 1 in the three datasets (TCGA-COAD dataset, GSE17536 dataset, and GSE17538 dataset) (Fig. 1D–F). The distribution of the two clusters in 3 MSI groups showed that there were more samples in clust2 with MSI-high than that in clust1 (Figure S2). PCA presented the different distribution of the two clusters in the three datasets (Figure S3). Of the distribution of the two clusters in different clinical features, we observed significant difference in T stage, N stage, M stage, and Event in TCGA- COAD dataset (Fig. 2).

**Table 1:** Genes associated to CRC prognosis using univariate Cox analysis

Genes	*P* value	HR	HR.95L	HR.95H
ADCY4	0.0312368184147054	1.71794194754031	1.04996352626844	2.81088291286421
ADCY5	0.0188279555148044	1.59123210899649	1.07995313326257	2.34456435813294
AKT3	0.0147860442279016	1.43237938385717	1.07294551758441	1.91222262982952
BCL10	0.0144005117806371	0.662634443586708	0.47657059505116	0.921341791514288
BID	0.0375619398603925	0.658648864512262	0.444365880377478	0.976263808451661
CASP10	0.00294943176675358	0.544913103771657	0.365171956038662	0.813124572552402
CASP7	0.0476711921188035	0.702719957694011	0.495606579534087	0.996385761072224
CCL28	0.0127097264874974	0.780860017161973	0.642798696309279	0.948574366910077
CXCL10	0.0165796018578295	0.850836151909923	0.745515137944138	0.971036160839339
CXCL11	0.0270332627274697	0.844380717306328	0.726806025020412	0.98097535135145
CXCL9	0.0450774127058808	0.869875505193357	0.759001350764162	0.996946044659301
DFFB	0.00655487194480544	0.44542235105924	0.248633886586798	0.797964724546425
FADD	0.0484652166986404	0.686376926819745	0.472309995080143	0.997466262789076
FAS	0.0238275679026361	0.728519168735319	0.553527029546879	0.958833355706709
GNG3	0.0111922683259024	2.36110967401405	1.21569164220528	4.58573432536715
GNG5	0.00118664607658216	0.559395123743022	0.393735272308479	0.794754563473058
MAPK11	0.0248005995950848	1.4730682662163	1.0503288939659	2.06595298805895
MAPK8	0.0298632318241826	0.527664037853723	0.296351798436873	0.939523020655482
NFAT5	0.0405181008773507	1.28472124649759	1.01087869326755	1.63274653249169
NGF	0.000401184995549343	2.57638832923589	1.52547502948327	4.35128513724103
PRKAR2A	0.0168537066614095	0.571122057771724	0.360761565966811	0.904143998818922
SPP1	0.013692506426785	1.12775756144109	1.02494837642101	1.24087919611001
STAT5B	0.0336369395673148	1.7034419473748	1.04209010399769	2.78451398486984
TRAF6	0.0176482130658922	1.7553010579624	1.1028690525217	2.7936968555234
XCL1	0.0161783822085616	1.80323097376245	1.11524355514241	2.91563392565051

### Differential score of cGAS STING-related pathways between the two clusters

**Fig. 3:**
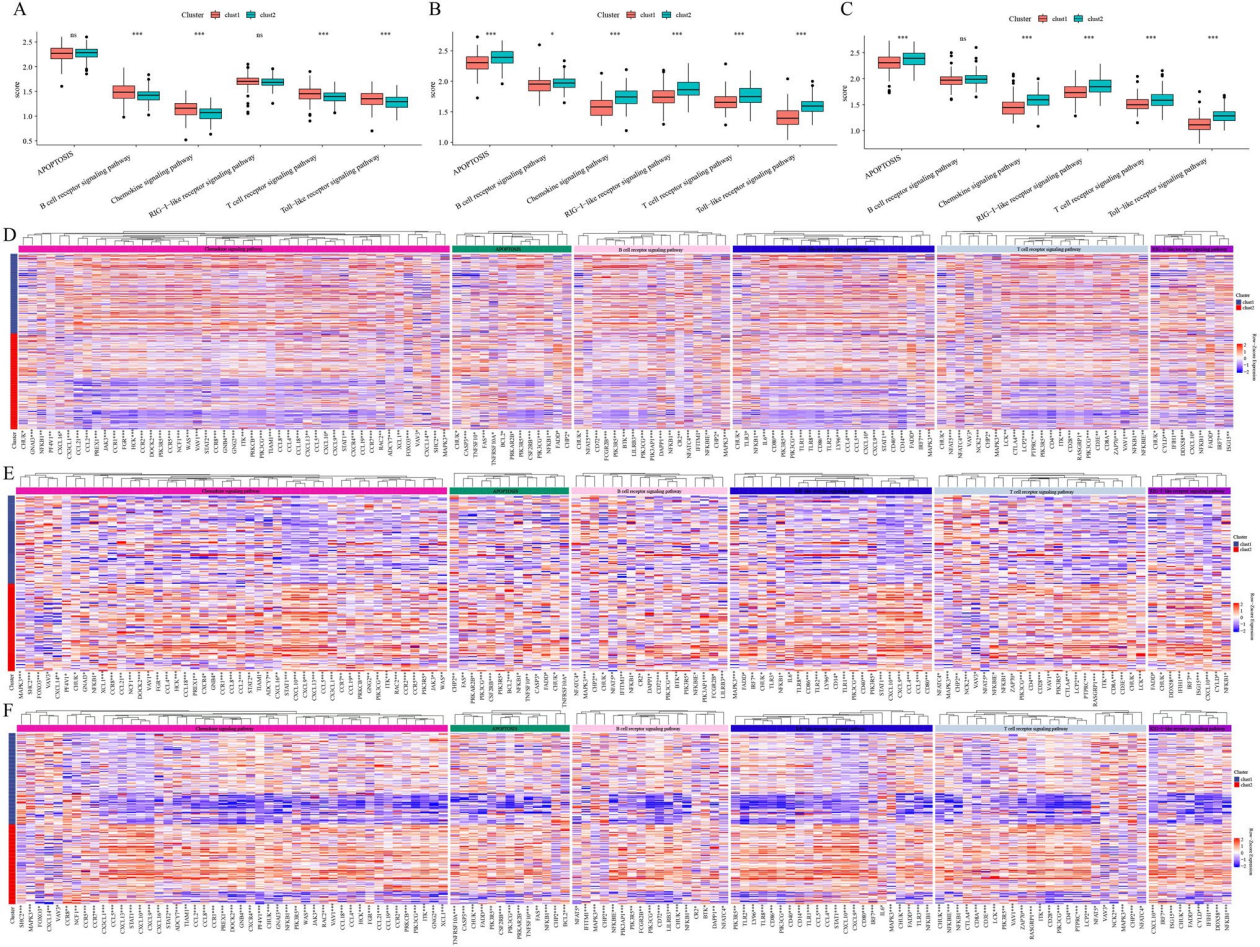
Immune pathways analysis in clusters. **A**–**C** Differences of six immune pathway score (apotosis, B cell receptor signaling pathway, Chemoking signaling pathway, RIG-I-like signaling pathway, T cell receptor signaling pathway, and Toll-like receptor signaling pathway) between two clusters respectively in TCGA-colorectal cancer, GSE17536 dataset, and GSE17536 dataset. **D**–**F** Differentially expressed gene between two clusters in six immune pathways respectively in TCGA-colorectal cancer, GSE17536 dataset, and GSE17536 dataset. Wilcoxon test was performed. *P < 0.05, ***P < 0.001

For each sample in TCGA-COAD dataset, GSE17536 dataset, and GSE17538 dataset, we calculated the enrichment score of six cGAS STING-related pathways including apoptosis, B cell receptor signaling pathway, Chemoking signaling pathway, RIG-I-like signaling pathway, T cell receptor signaling pathway, and Toll-like receptor signaling pathway using ssGSEA. The results showed significant differences in B cell receptor signaling pathway, Chemoking signaling pathway, T cell receptor signaling pathway, and Toll-like receptor signaling pathway between the two clusters in TCGA-COAD dataset (Fig. 3A). The six cGAS STING-related pathways all showed significant difference between the two clusters in GSE17536 dataset (Fig. 3B) in terms of apoptosis, Chemoking signaling pathway, RIG-I-like signaling pathway, T cell receptor signaling pathway, and Toll-like receptor signaling pathway between the two clusters in GSE17538 dataset (Fig. 3C). Next, the expressions of genes in six cGAS-STING-related pathways between the two clusters were calculated in three datasets; genes showing significant differences in the three datasets are shown in Fig. 3D–F.

### Differential genomic features between the two subtypes

**Fig. 4:**
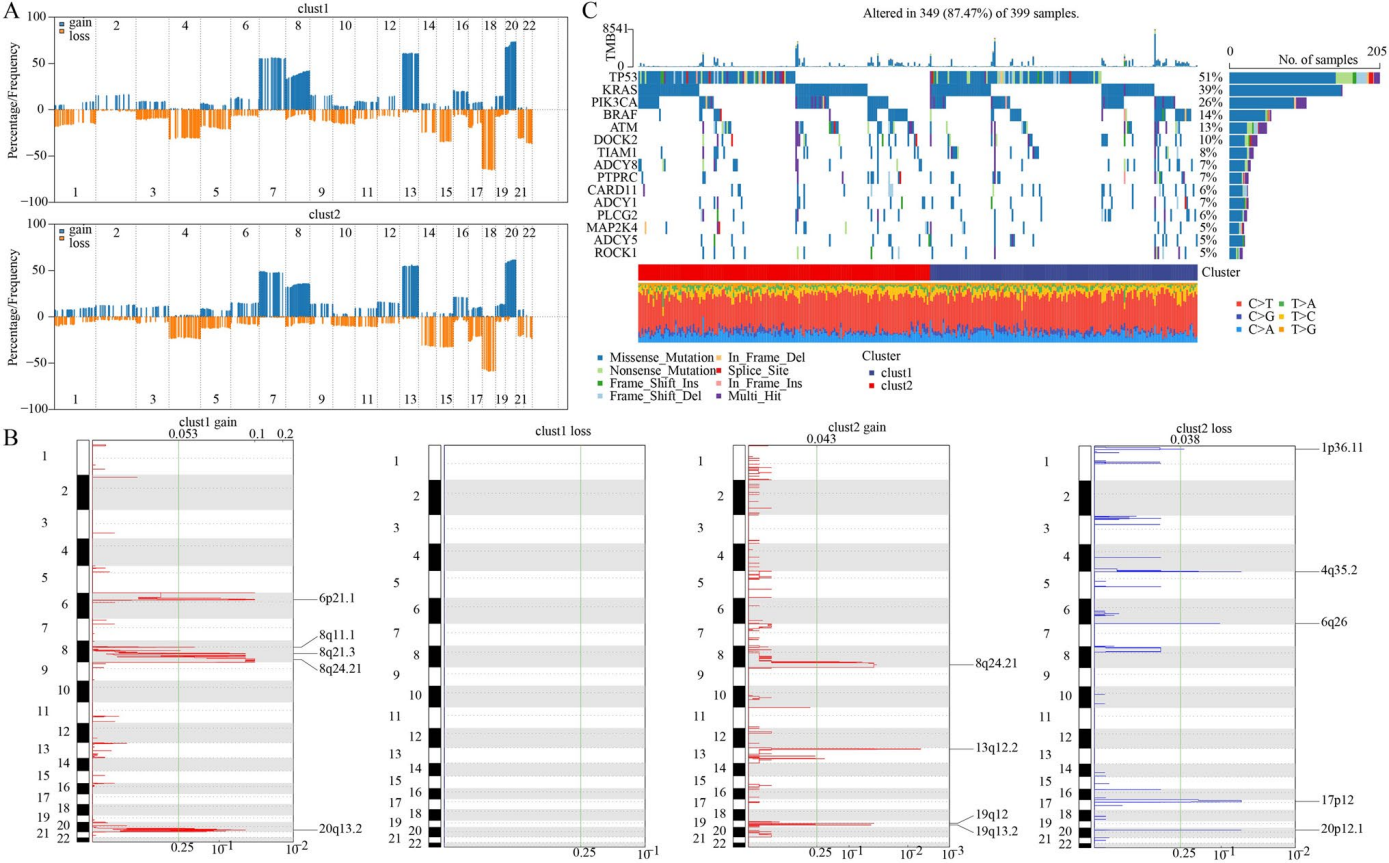
Genomic analysis. **A** Copy number variation in two clusters. **B** The details of copy number amplification and deletion in two clusters. **C** Top 15 single-nucleotide variant genes in two clusters

To understand the genomic features of three subtypes, we applied gistic2 software to analyze the CNV data and visualize the CNVs of 22 chromosomes (Fig. 4A). Although similar CNV patterns were shown in the two clusters, still a number of significantly differential CNVs were identified among them. Clust1 had the most amplified CNVs and clust2 had the most loss CNVs (Fig. 4B). In addition, we screened the top 15 mutated genes within the six cGAS-STING-related pathways based on SNV data using maftools software (Fig. 4C). Statistical analysis showed that the mutation frequency of these genes was not statistically significant between the two clusters (Table 2). *TP53* was the top mutated gene of which missense mutations contributed the most of SNVs.

**Table 2:** The gene mutation in two subtypes

Genes	Clust1	Clust2	*P* value
ADCY1	16	13	0.706623340047281
ADCY5	13	9	0.518417617226903
ADCY8	13	20	0.220822426135479
ATM	29	40	0.171185213186279
BRAF	32	28	0.689149767784179
CARD11	14	12	0.842807287529148
DOCK2	27	24	0.773601229164655
KRAS	73	83	0.381404600427855
MAP2K4	9	13	0.398923681573876
PIK3CA	60	63	0.772386889671076
PLCG2	15	14	1
PTPRC	17	14	0.715475923779207
ROCK1	15	9	0.301092609353931
TIAM1	23	18	0.523349528896189
TP53	109	104	0.754984323750719

### Differential TME among the two clusters

**Fig. 5:**
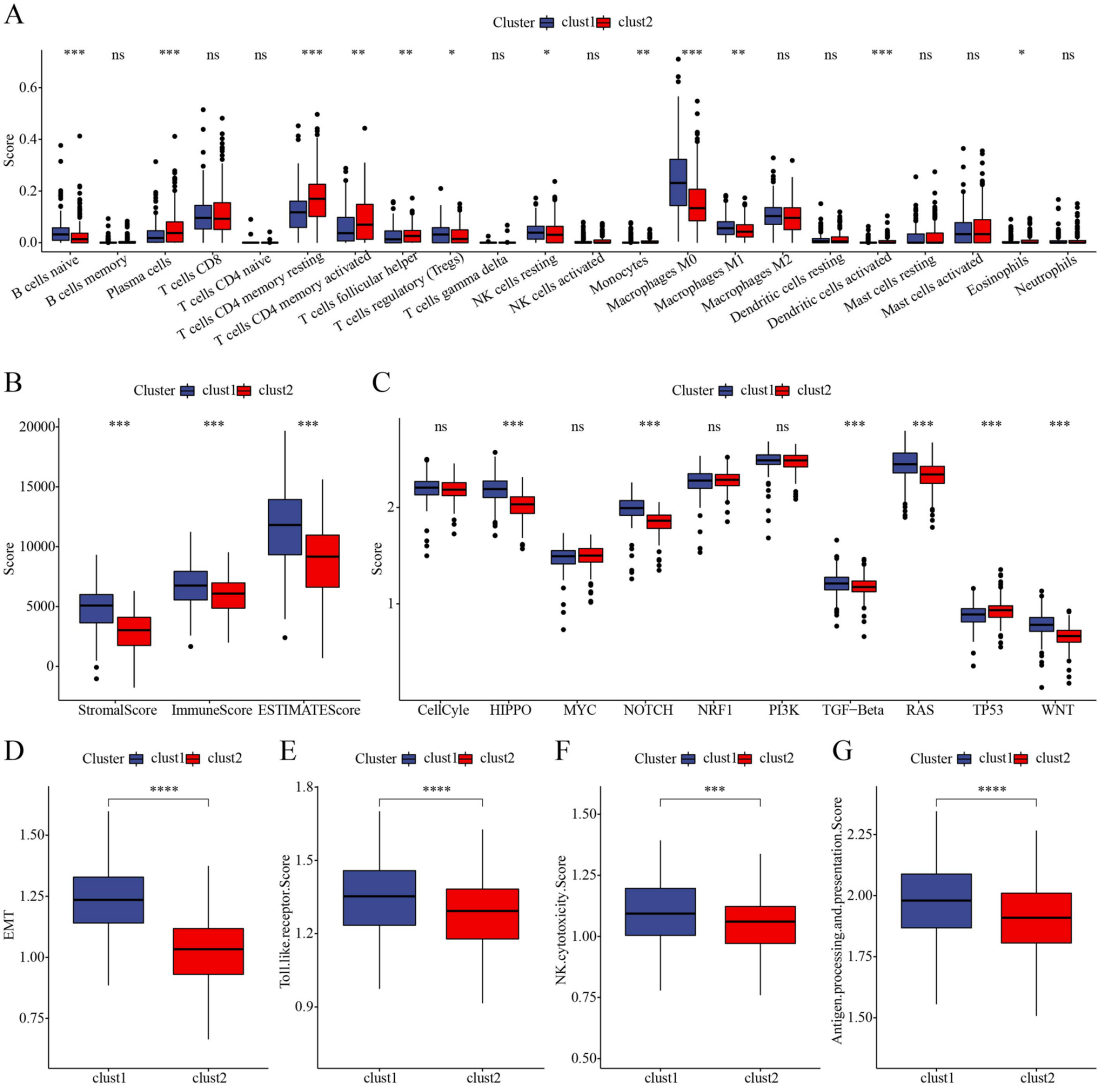
Immune cells analysis in clusters. **A** Differences of 22 type immune cell score between two clusters in TCGA-colorectal cancer. **B** Differences of stromalScore, immuneScore, and ESTIMATEscore between two clusters in TCGA-colorectal cancer. **C** Differences of 10 oncogenic pathway score between two clusters in TCGA-colorectal cancer. **D**–**G** Score differences of EMT, Toll-like receptor signaling pathway, natural killer (NK) cell–mediated cytotoxicity and antigen processing and presentation between two clusters in TCGA-colorectal cancer. Wilcoxon test was performed. *P < 0.05, **P < 0.01, ***P < 0.001

Next, we estimated the proportion of 22 immune-related cells in the two clusters in TCGA-COAD dataset. Twelve out of 22 immune-related cells showed a significant difference on the proportion between two clusters (Fig. 5A). Especially, T cell CD4 memory resting and M0 macrophages accounted for a relatively high proportion among 22 immune-related cells. Clust1 had the most proportion of M0 macrophages, while clust2 had the most proportion of T cell CD4 memory resting (Fig. 5A). Surprisingly, clust1 had a higher score of stromal, immune, and ESTIMATE score (Fig. 5B). We then assessed the enrichment of 10 oncogenic pathways (Sanchez-Vega et al. 2018), and observed 6 out of 10 oncogenic pathways were differentially enriched between the two clusters (Fig. 5C). Analysis of enrichment score of EMT showed that the EMT score was higher in clust1 than that in clust2 (Fig. 5D). As M0 macrophages were identified to be significantly differentially enriched between the two clusters, we further assessed the immune regulation related to macrophages. We selected three pathways related to macrophages from MSigDB including Toll-like receptor signaling pathway, NK cell–mediated cytotoxicity, and antigen processing and presentation. GSEA revealed that the three pathways were the most activated in clust1 (Fig. 5E–G), which was in accordance to the highest proportion of macrophages in clust1.

### Identification of DEGs between the two clusters

**Fig. 6:**
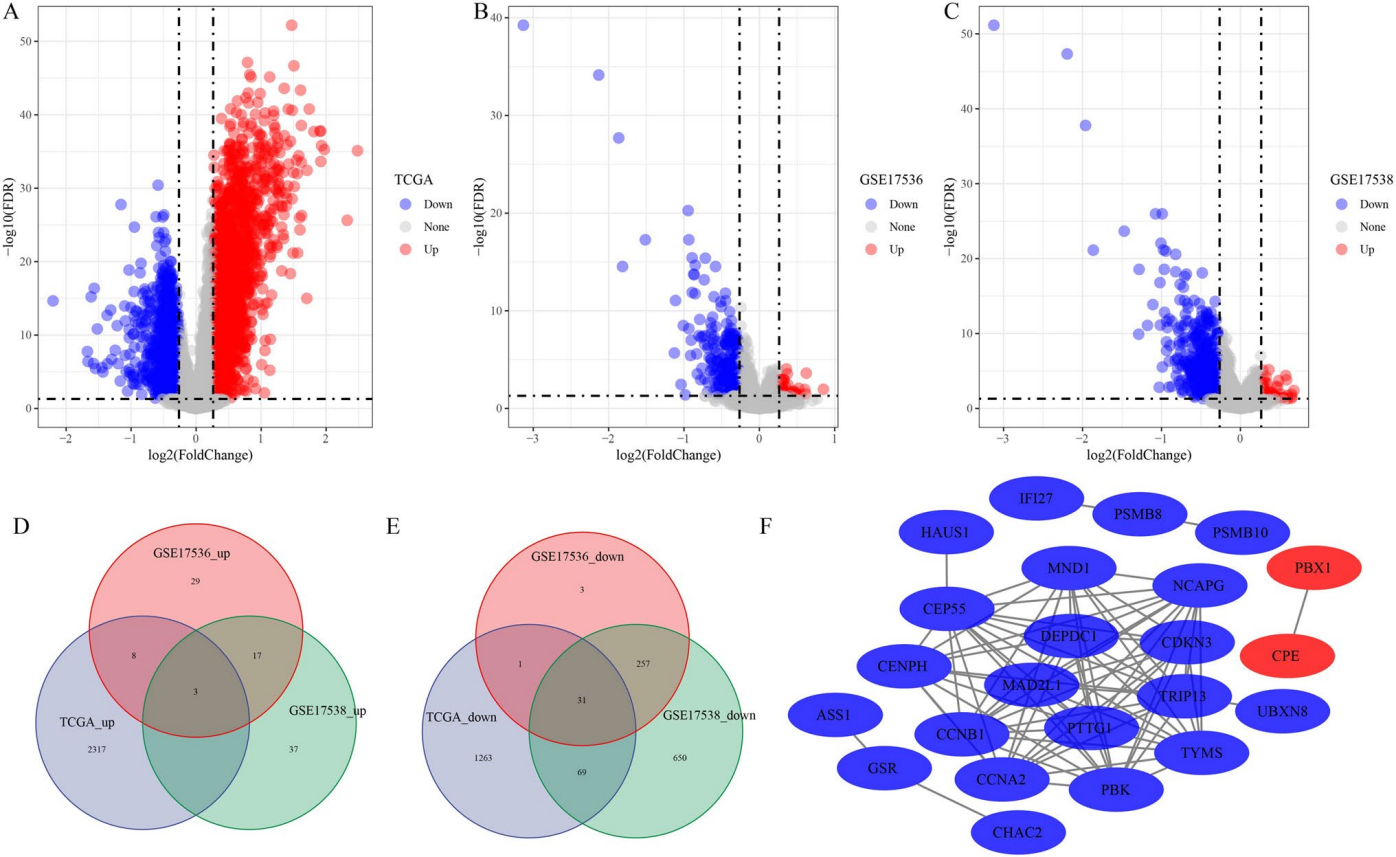
Identification of DEGs. **A**–**C** Identification of differentially expressed gene between two clusters respectively in TCGA-colorectal cancer, GSE17536 dataset, and GSE17538 dataset. **D**–**E** The Venn diagram of differentially expressed upregulated genes and differentially expressed down-regulated genes among TCGA-colorectal cancer, GSE17536 dataset, and GSE17538 dataset. **F** Protein–protein interaction (PPI) analysis of differentially expressed genes

Limma package was used to screen DEGs between the two clusters in TCGA-COAD dataset, GSE17536 dataset, and GSE17538 dataset. In TCGA-COAD dataset, 1364 upregulated genes and 2328 downregulated gene were identified between the two clusters (Fig. 6A). Fifty-seven upregulated genes and 292 downregulated gene were identified in GSE17536 dataset (Fig. 6B). Fifty-seven upregulated genes and 1007 downregulated gene were identified in GSE17538 dataset (Fig. 6C). Furthermore, 3 upregulated and 31 downregulated genes were screened in three datasets (Fig. 6D, E). Based on the 34 genes, STRING (https://cn.string-db.org/) was used to predict their interactions and we found 23 interacted genes visualized by the CytoScape (Fig. 6F). The functional enrichment analysis results indicated that those 34 genes were enriched in cell cycle pathway and chromosomal region (Figure S4).

### Identification of seven cell types based on scRNA-seq data

**Fig. 7:**
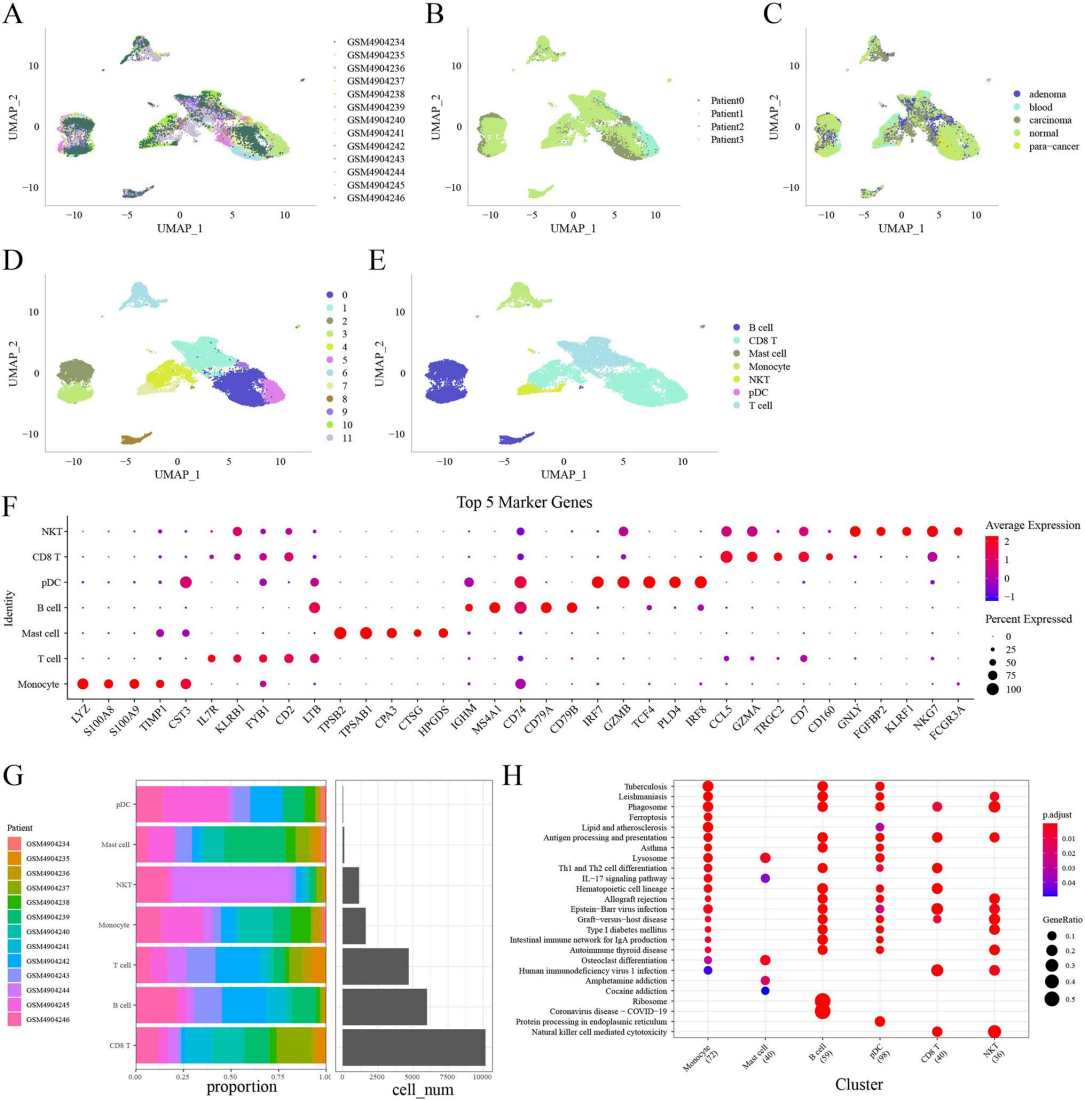
Single-cell analysis of GSE161277 dataset. **A**–**E** T-SNE plots of 26188 single cells grouped by samples (**A**), patients (**B**), tissues (**C**), subpopulation (**D**), and immune cells (**E**). **F** The top 5 DEGs (markers) of seven immune cell types. **G** The cell counts of different cell types and the distribution of samples. **H** Significantly enriched pathways of six immune cell types

To further verify the reliability of the two clusters based on cGAS-STING-related pathways, scRNA-seq data of COAD (GSE161277 dataset) was used to distinguish malignant and non-malignant cells. Single-cell data analysis obtained 26,961 cells. This conformed to the criteria that each gene was expressed in at least 3 cells, each cell expressed at least 250 genes, genes expressed in each cell were greater than 100 and less than 7000, and the mitochondrial content was less than 25%, and UMI of each cell was at least greater than 100 but less than 5000 (Figure S5A-C). By using PCA analysis (dim = 40), the cells were clustered into 29 clusters (resolution = 0.9) and CD45 marker was used to identify immune cells with a total number of 23,803 cells (Figure S5D). Based on above results, 29 clusters were reduced to 12 clusters under dim = 30 and resolution = 0.2 (Figure S5E). T-SNE plots were grouped by different samples, patients, tissues, and subpopulation (Fig. 7A–D). Seven cell types were annotated (Fig. 7E). The top 5 DEGs among the 7 cell types were identified (Fig. 7F). The CD8 T cells, B cells, and T cells were the top 3 (Fig. 7G). KEGG analysis indicated that 25 pathways were significantly enriched to 7 cell types and many of them were related to immunity (Fig. 7H).

### Malignant cells were richer in clust1

**Fig. 8:**
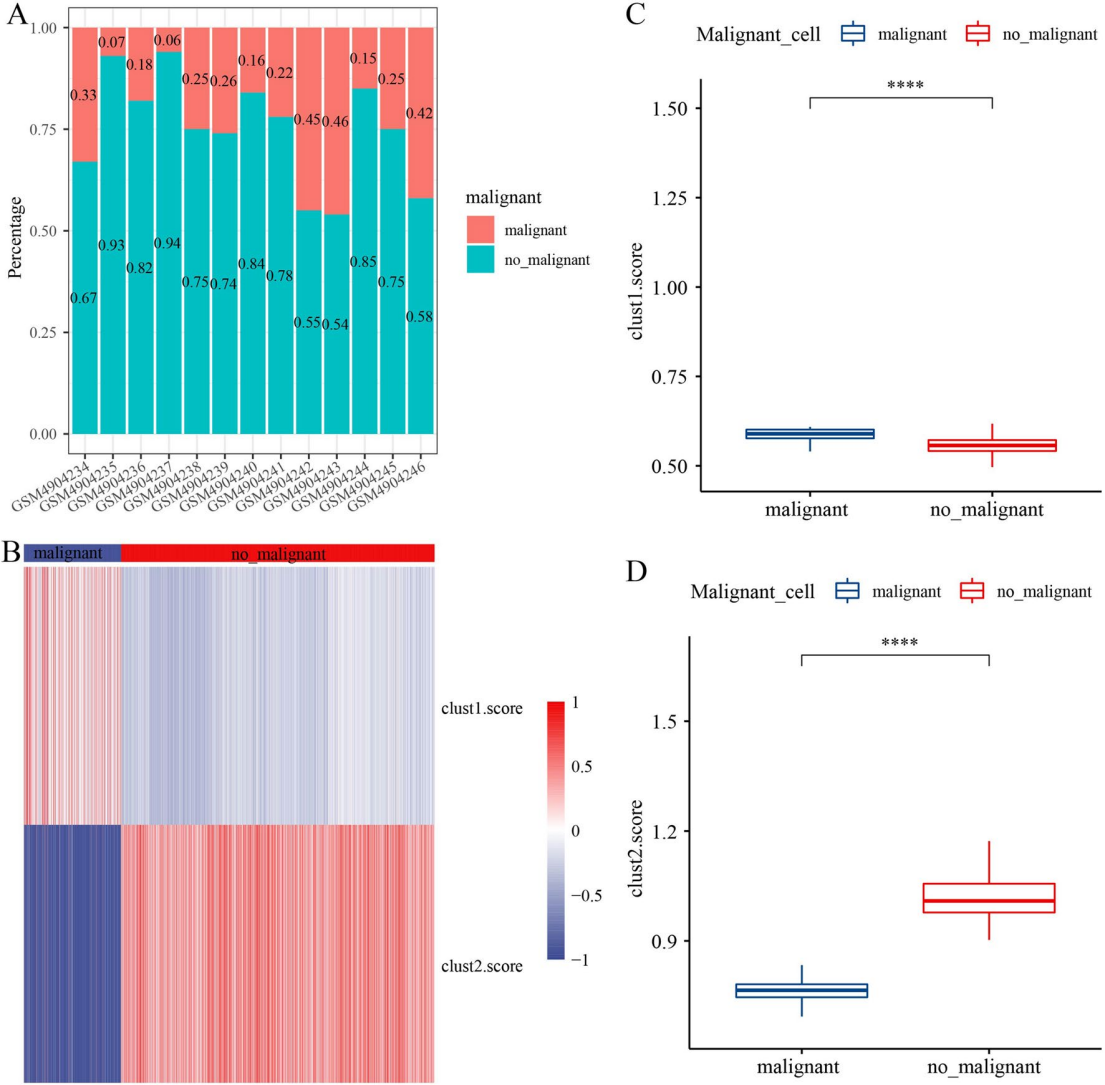
The malignant cells in clusters. **A** The distribution of malignant cells in 13 samples. **B** The distribution of clust1 score and clust2 score in malignant and non-malignant cells. **C** The distribution of clust1 score in malignant and non-malignant cells. **D** The distribution of clust2 score in malignant and non-malignant cells. Wilcoxon test was performed. ****P < 0.0001

To further validate that the enrichment score of the immune pathway for the poor prognostic clust1 isoform obtained by bulk RNA seq analysis was lower than that of the favorable prognosis in clust2, the Copykat package was used to predict the changes of the CNV of 7 cells types cells for distinguishing the tumor cells and normal cells in each sample. The percentage of those cells in 13 samples were shown (Fig. 8A). ssGSEA was used to calculate enrichment score of 3 upregulated genes (clust1) and 31 downregulated genes (clust2) in malignant and non-malignant cells, respectively (Fig. 8B, Figure S6). Significantly differential enrichment of these genes was exhibited in two groups, with the clust1 scoring higher in malignant cells and clust2 scoring higher in no-malignant cells (Fig. 8C–D). The result demonstrated that the molecular subtyping based on cGAS-STING-related pathways was reliable.

### Metabolism of malignant cells were associated with TME

**Fig. 9:**
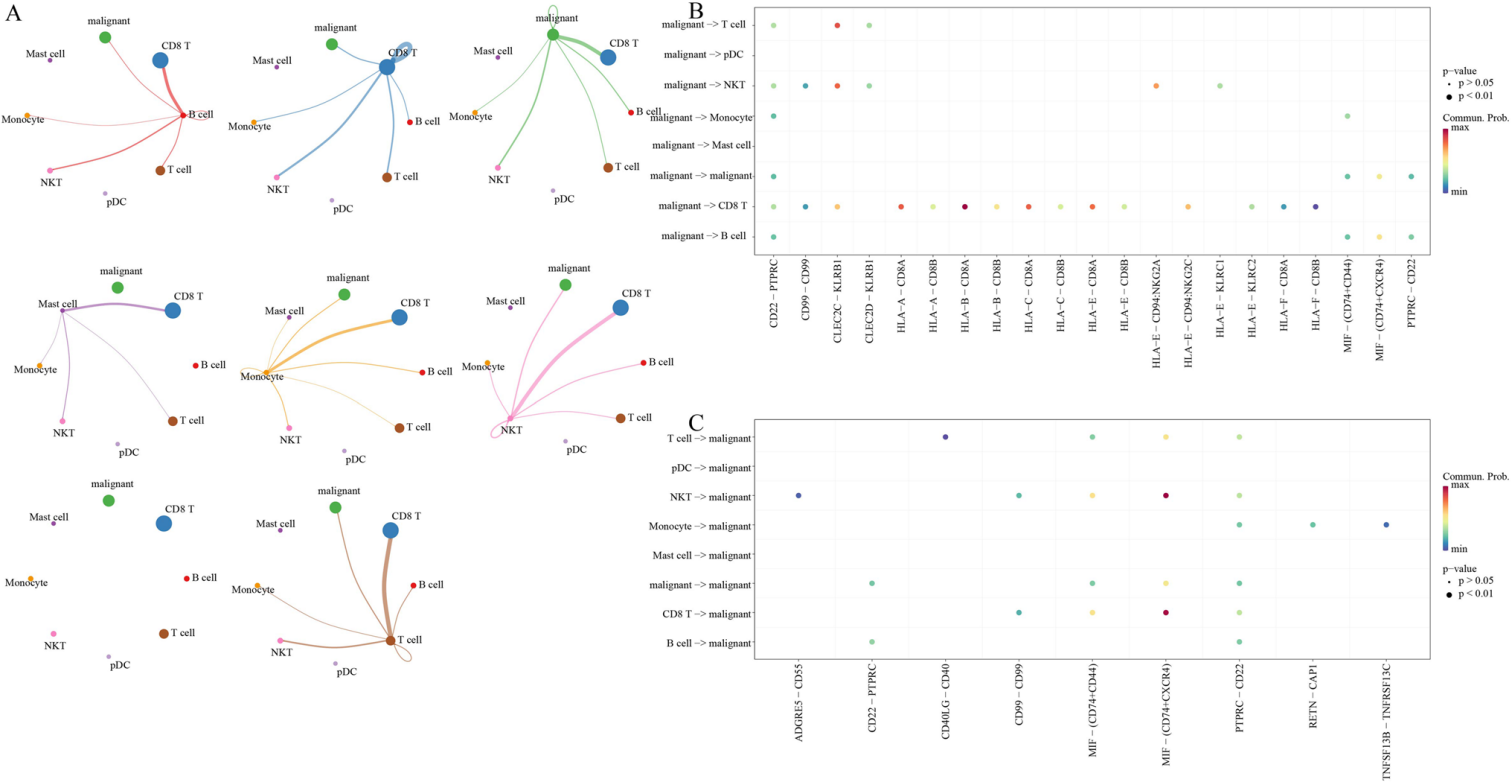
The correlation of malignant cells and tumor microenvironment. **A** The number of ligand receptor pairs acts as the strength of the cell–cell interaction. **B** Key ligand receptor pairs between malignant cells and immune cells. **C** Key ligand receptor pairs between immune cells and malignant cells

Cellchart package was used for cell-to-cell binding receptor analysis of 23,803 cells, including 6375 malignant cells and 17,428 normal immune cells. The number of binding receptors was used as the strength of cell–cell interaction. There was strong interaction between malignant cells and 6 cell types (Fig. 9A). The dot plot of the interaction of the key coordination receptor pairs in malignant cells-immune cells and immune cells-malignant cells revealed a close interaction between malignant cells and immune cells (Fig. 9B, C). Further analysis showed that five coordination receptors (MIF-(CD74 + CXCR4), MIF-(CD74 + CD44), CD99-CD99, CD22-PTPRC, PTPRC-CD22) played an important role in malignant cell-immune cell communication and immune cell-malignant cell communication (Figure S7).

**Fig. 10:**
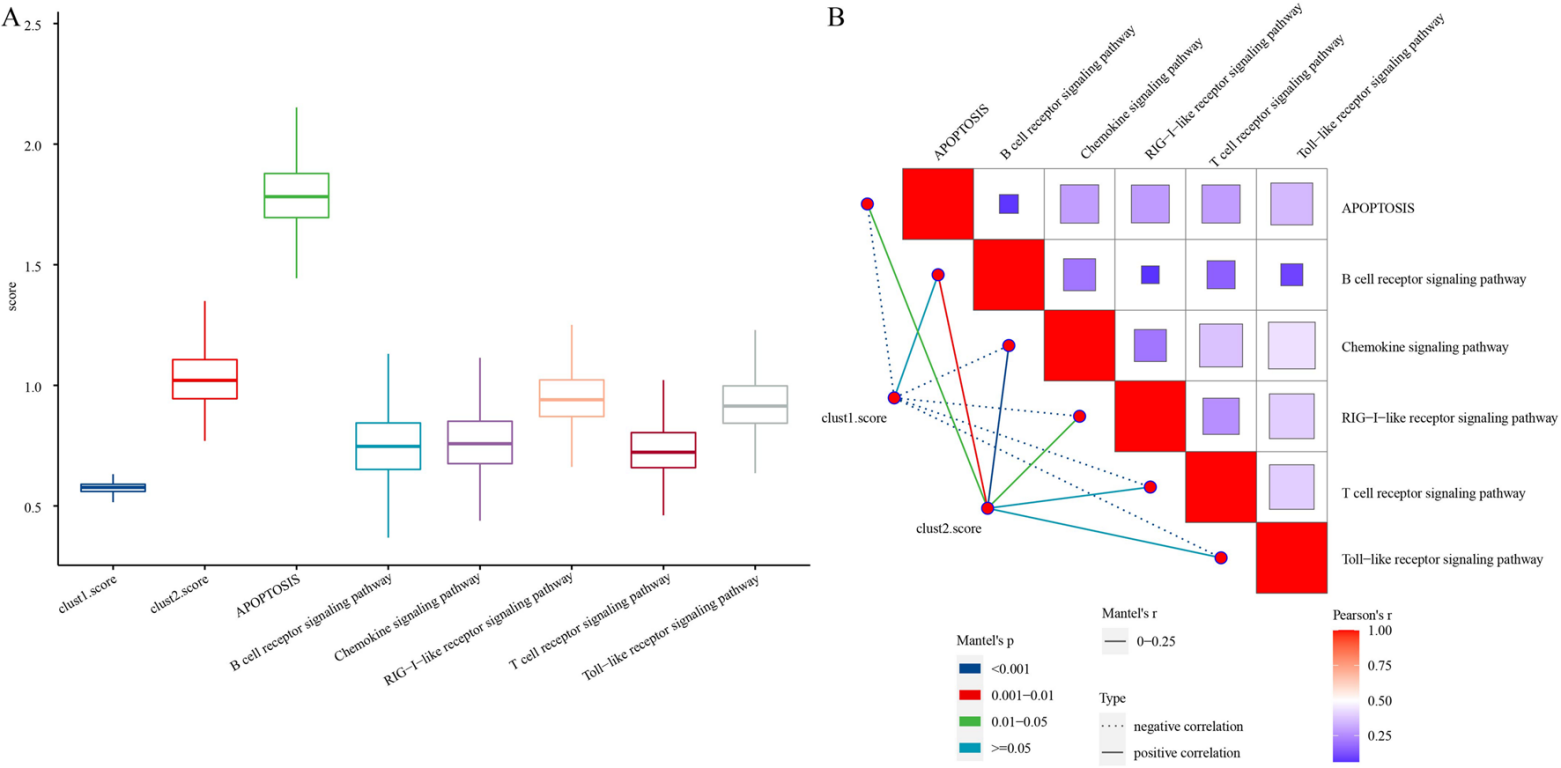
The correlation of malignant cells and cGAS-STING pathways. **A** Scores of malignant cells in six immune-related pathways and clusters. **B** In malignant cells, correlation analysis of clusters score and six cGAS-STING pathways

To better analyze the relationship between malignant cells and the six immune-related pathways, the score of 6375 malignant cells in 6 immune-related pathways and clust1 and clust2 was determined by ssGSEA method. The results showed that clust2.score was much higher than clust1.score in malignant cells. Among the six immune-related pathways, chemokine signaling pathway had the highest score and RIG I like receptor signaling pathway had the lowest score (Fig. 10A). Pearson correlation analysis on the six immune-related pathways in relation to clust1.score and clust2.score showed that clust2.score was significantly positively correlated with immune-related pathways, while clust1.score was negatively correlated with immune-related pathways (Fig. 10B).

## Discussion

In the present study, we determined 27 key prognosis genes from the six immune pathways by analyzing expression data. Initially, patients in the TCGA-colorectal cancer cohort were divided into two clusters using ConsensusClusterPlus clustering based on 27 genes. We noticed that the samples in Clust2 were significantly correlated with favorable prognosis, low immune infiltration level, less macrophages, and low EMT score. Then 34 important DEGs were identified between clust1 and clust2, and 40 genes showed close interactions through PPI analysis. Furthermore, scRNA analysis confirmed that immune enrichment score of clust1 was lower than that of clust2.

The cGAS-STING pathway can be activated by radiation-induced DNA damage and because of its important role in anti-cancer immunity activation, to increase its activation in cancer cells could provide significant therapeutic benefits for patients (Wan et al. 2020). Studies have found that mice with STING defects tend to develop several types of cancer and have a low survival rate under the burden of tumors, while STING stimulation can induce strong immunity to tumors (Ohkuri et al. 2014; Kitajima et al. 2019). cGAS-STING promotes cancer cell senescence via the p53-p21 pathway (Kitajima et al. 2019). cGAS-STING-mediated autophagy contributes to autophagy death during mitotic crises to avoid transformation of cancer cells (Nassour et al. 2019). Activation of endothelial cell STING in tumor microenvironment can promote tumor vascular remodeling and may have a positive effect on tumor regression (Yang et al. 2019). Currently, studies (Marill et al. 2019; Wei et al. 2021) showed that cGAS-STING pathway was activated in colorectal cancer. In this study, we were the first to study the immune heterogeneity of CGAS-STING pathway in colorectal cancer, and we divided colorectal cancer samples into two subtypes with significant characteristic differences.

Another important finding in the present study was that cGAS-STING pathway–related clusters were associated with various immune cell infiltration levels in colorectal cancer. Macrophages could display anti-tumor M1 and pro-tumor M2 phenotypes, and high density of M1 macrophages was associated with better overall survival in colorectal cancer (Liu et al. 2020). Macrophages, such as macrophage antigen presentation and Toll-like receptors, play an important role in immune regulation. Macrophages show the presence of Fc receptors. Tumor cells can be killed by specific antibody-dependent cellular cytotoxicity (natural killer cell–mediated cytotoxicity) (de Taeye et al. 2020). Our results demonstrated a significant correlation between the cGAS-STING pathway–related clusters and the infiltration of immune cells, such as macrophages, Toll-like receptor, antigen and processing and presentation, and NK cytotoxicity. This indicated that cGAS-STING pathway–related clusters may be a new and effective classification system for clinical colorectal cancer patients and can reflect the immune status.

The present study had some limitations. Firstly, all the data in the present study were obtained from online databases; hence, further studies involving larger sample sizes, as well as in vitro and in vivo experiments, are needed to confirm our results. Secondly, we did not analyze the potential mechanisms of cGAS-STING pathway members in colorectal cancer. Future studies should be performed to investigate the detailed mechanism between cGAS-STING pathway members and colorectal cancer.

## Conclusions

In summary, we developed cGAS-STING pathway–related subtypes for colorectal cancer, which could be used to predict prognosis. We found that the underlying molecular mechanisms may affect immune-related biological processes, which may provide novel insights into the relationship between colorectal cancer and tumor immune infiltration.

## Supplementary Information

Below is the link to the electronic supplementary material.

Supplementary file1 (JPG 594 kb)

Supplementary file2 (JPG 481 kb)

Supplementary file3 (JPG 299 kb)

Supplementary file4 (JPG 1021 kb)

Supplementary file5 (JPG 1833 kb)

Supplementary file6 (JPG 587 kb)

Supplementary file7 (JPG 89 kb)

## Data Availability

All data generated or analyzed during this study are included in this published article.
