# Supplementary figures and images for: Integration of bulk RNA sequencing data and single-cell RNA sequencing analysis on the heterogeneity in patients with colorectal cancer
Source: Funct Integr Genomics. 2023 Jun 24;23(3):209. doi: 10.1007/s10142-023-01102-3 (PMC10290593; doi:10.1007/s10142-023-01102-3)

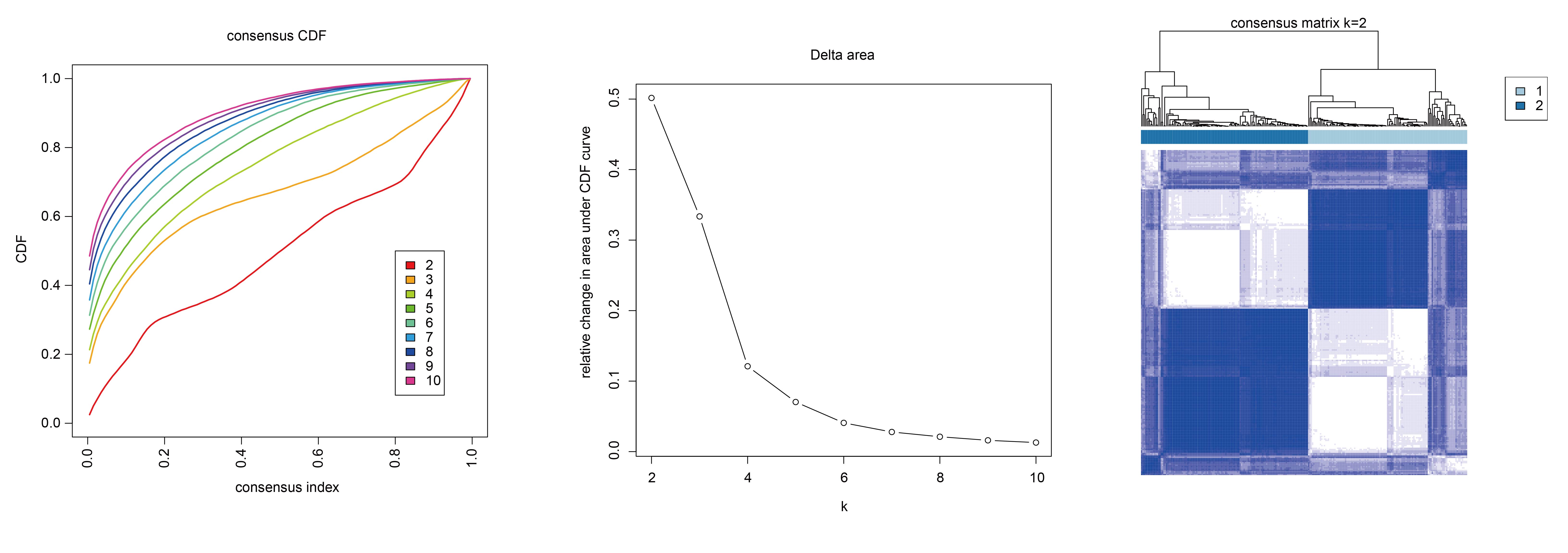

Supplement: Supplementary file 1 — Supplementary file1 (JPG 594 kb) [file 10142_2023_1102_MOESM1_ESM.jpg]

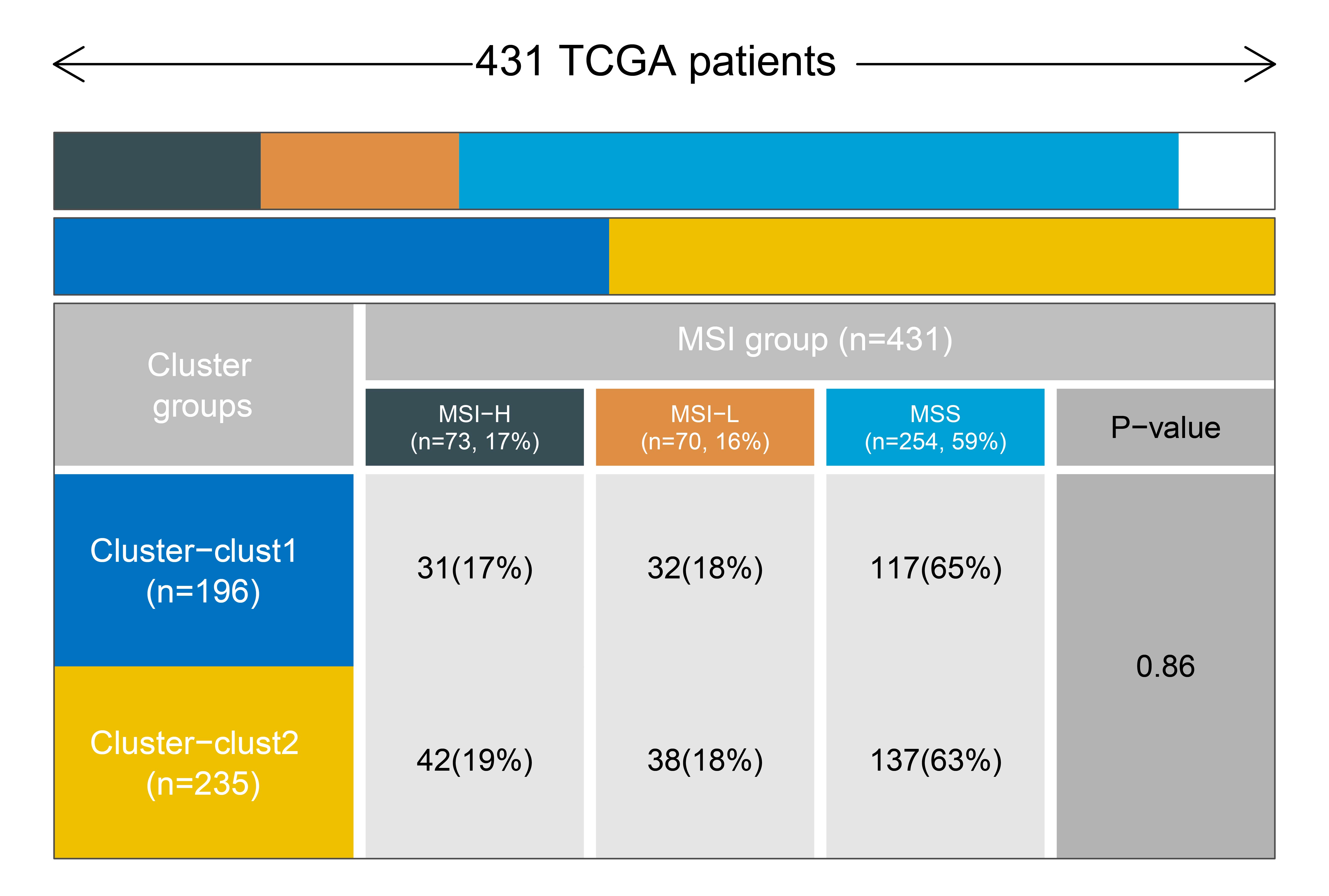

Supplement: Supplementary file 2 — Supplementary file2 (JPG 481 kb) [file 10142_2023_1102_MOESM2_ESM.jpg]

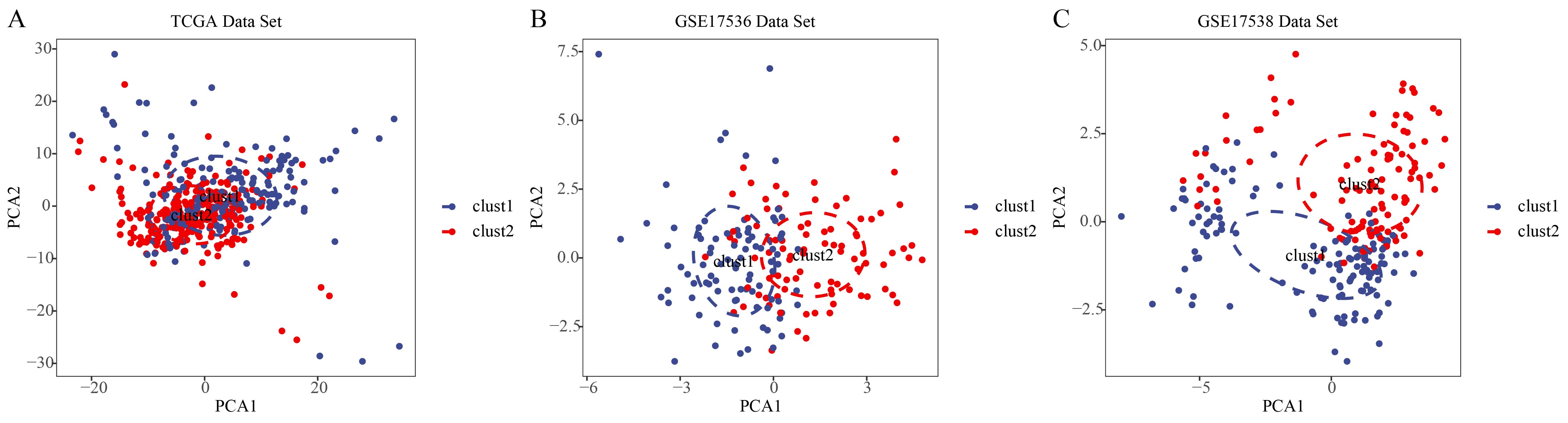

Supplement: Supplementary file 3 — Supplementary file3 (JPG 299 kb) [file 10142_2023_1102_MOESM3_ESM.jpg]

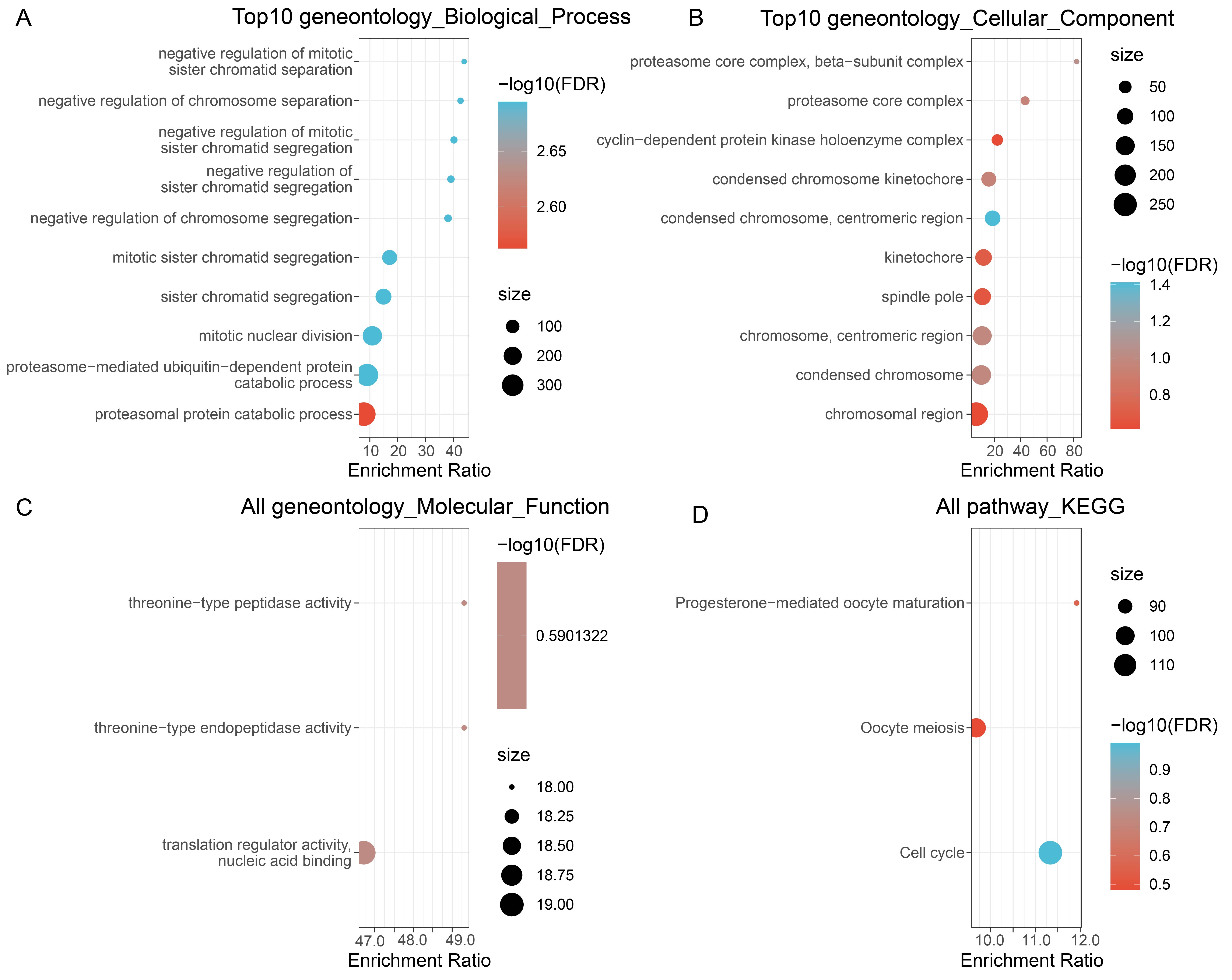

Supplement: Supplementary file 4 — Supplementary file4 (JPG 1021 kb) [file 10142_2023_1102_MOESM4_ESM.jpg]

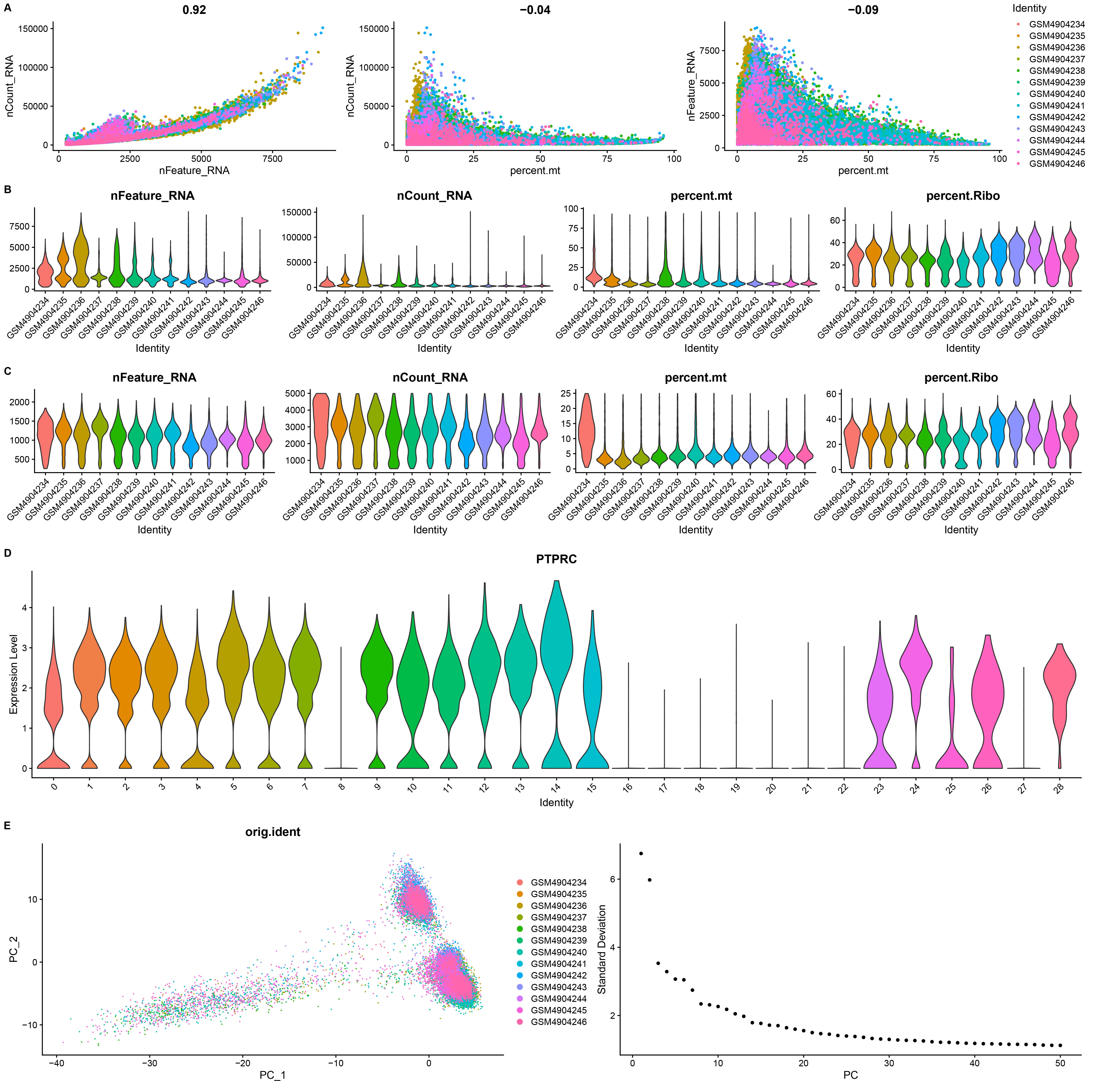

Supplement: Supplementary file 5 — Supplementary file5 (JPG 1833 kb) [file 10142_2023_1102_MOESM5_ESM.jpg]

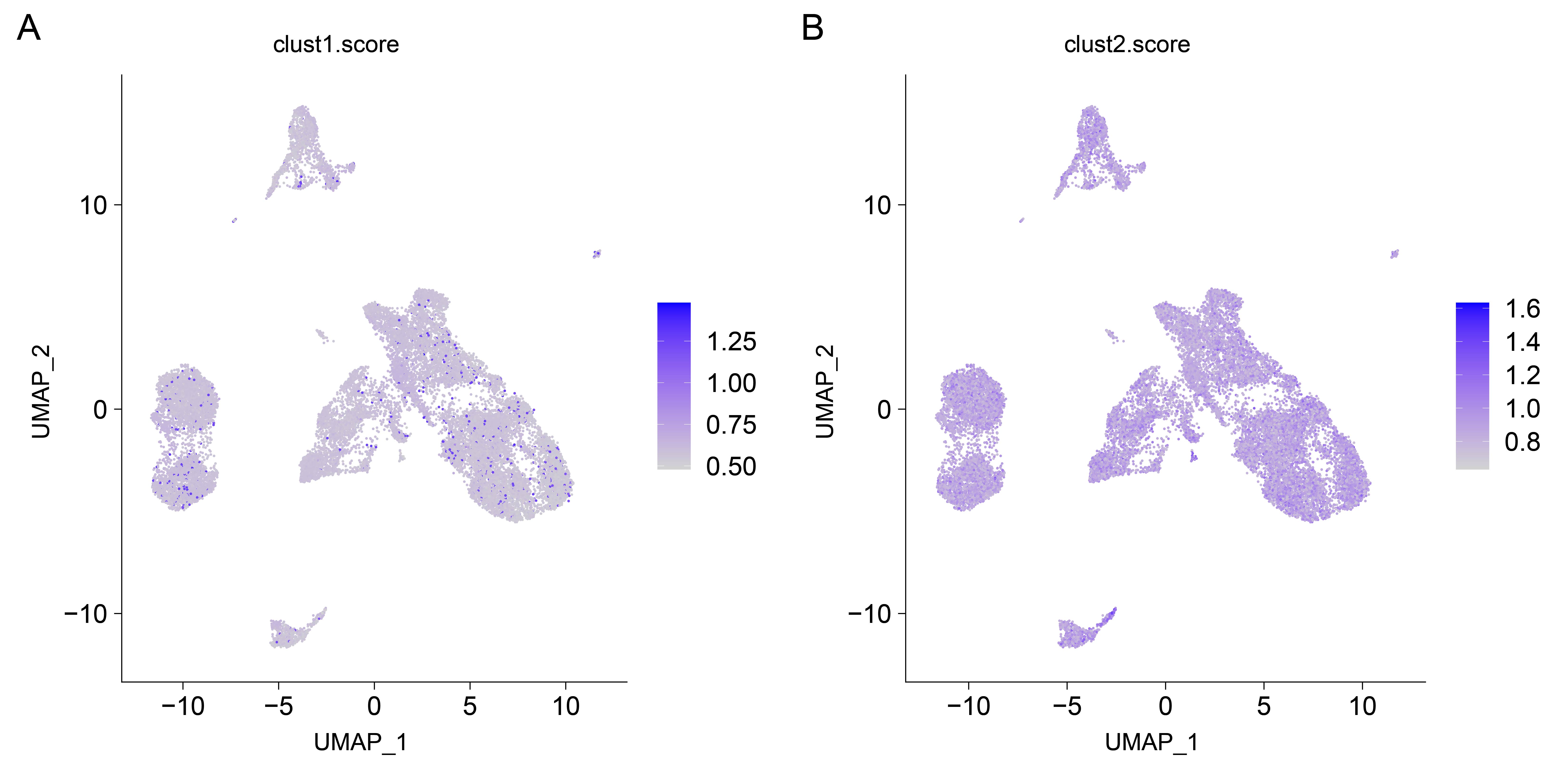

Supplement: Supplementary file 6 — Supplementary file6 (JPG 587 kb) [file 10142_2023_1102_MOESM6_ESM.jpg]

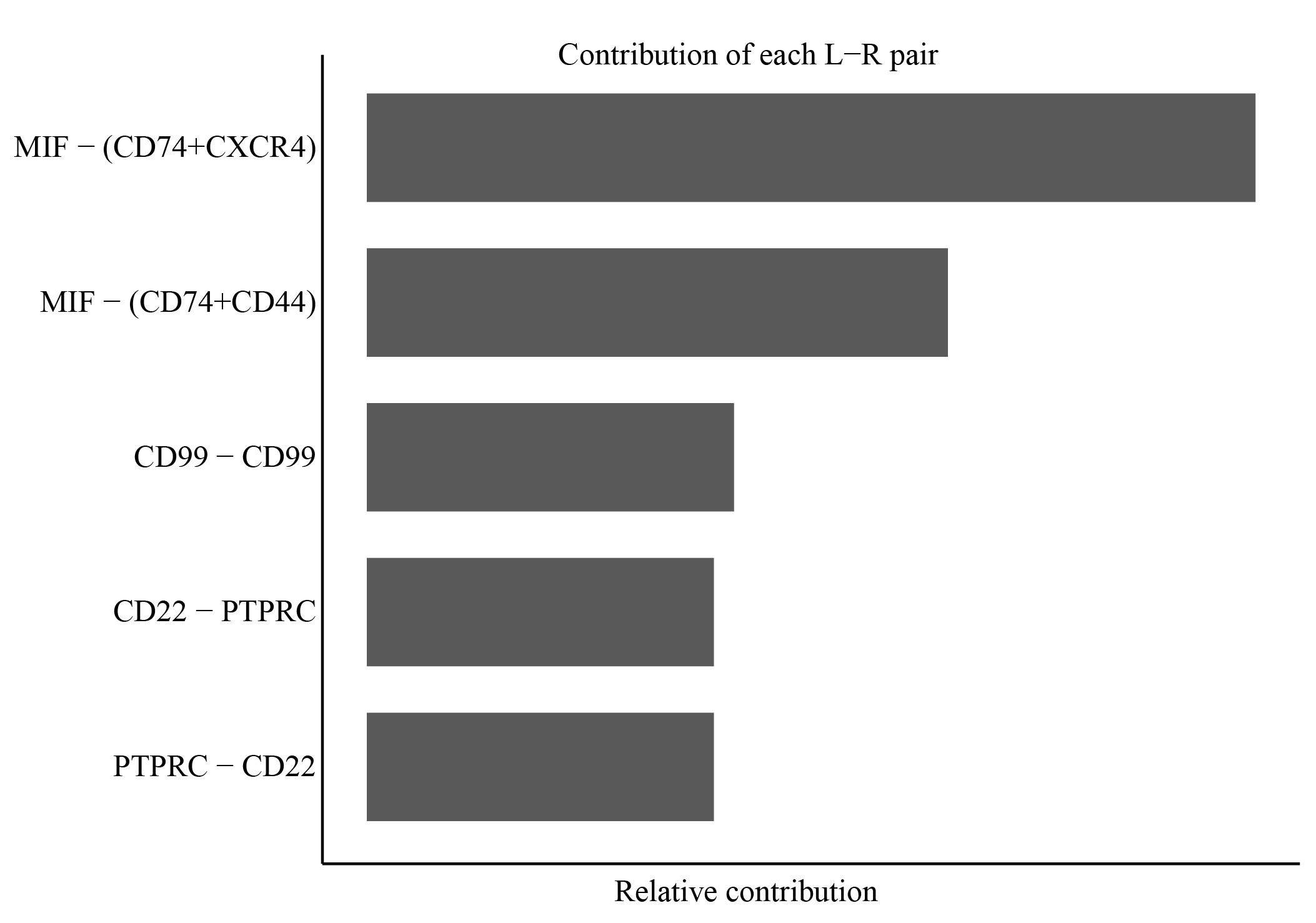

Supplement: Supplementary file 7 — Supplementary file7 (JPG 89 kb) [file 10142_2023_1102_MOESM7_ESM.jpg]
